# Nocardiosis: A two-center analysis of clinical characteristics

**DOI:** 10.3389/fmed.2022.996442

**Published:** 2022-11-16

**Authors:** Lumin Wang, Yijiao Xu, Zhisheng Chen, Weiwen Jiang, Xiong Xiao, Yun Shen, Yanrong Ye

**Affiliations:** ^1^Xiamen Branch, Zhongshan Hospital, Fudan University, Xiamen, China; ^2^Zhongshan Hospital, Fudan University, Shanghai, China

**Keywords:** *Nocardia*, nocardiosis, clinical characteristics, immunosuppressed, two-center retrospective study

## Abstract

**Objective:**

The objective of the present study was to describe and analyze the clinical characteristics of nocardiosis.

**Materials and methods:**

We described and analyzed the clinical characteristics of nocardiosis cases from two centers over the past 5 years from the following aspects: age and sex, *Nocardia* species, sites of *Nocardia* infection, test specimens, detection methods, concurrent pathogens, symptoms, imaging features, co-conditions, drug susceptibility tests, antibiotic therapy/duration, outcomes, and follow-up.

**Results:**

The median age of the 19 cases was 64 years, with an interquartile range (IQR) of 56–68 years. Eight cases (42.1%) were immunocompromised [those who had been on corticosteroid use (62.5%), those who had used immunosuppressants (50.0%), or those who had suffered from chronic nephrosis (37.5%) or diabetes mellitus (DM) (25.0%)]. The plethora of comorbidities of these cases included diabetes (10.5%), chronic kidney disease (CDK) (15.8%), chronic lung disease (36.8%), and rheumatic diseases (10.5%). Cough and expectoration (73.7%) was the most common symptom of nocardiosis. The respiratory tract (89.5%) was the most common site of the clinical disease. Nearly half (9 cases, 47.3%) of these patients had concurrent infections. The most common *Nocardia* isolation site was the respiratory tract (73.7%). All patients were given antibiotic therapies, out of whom as many as 63.6% of patients were treated with two concurrent antimicrobial agents, 15.8% of patients were treated under monotherapy and 21.1% of patients were treated with three or more concurrent antimicrobial agents.

**Conclusions:**

An uncommon life-threatening infection, nocardiosis, affects those patients with structural lung disease or immunosuppression. Although nocardiosis is capable of progressing into a serious and metastatic disease, early recognition and prompt treatment usually result in successful outcomes benefitting the patient.

## Introduction

*Nocardia* is an aerobic Gram-positive filamentous bacterium that belongs to the actinomycetes group. *Nocardia* exists in soil, persists in decomposing vegetation and some other organic matter thrives in fresh water and subsists even in salt water (1). *Nocardia* is also known to give rise to opportunistic infections, referred to as nocardiosis. The infection is usually caused by exogenous inhalation or by a direct invasion of the injured skin. Pulmonary infection is often caused by inhaling broken hyphae. The individuals who are most vulnerable to *Nocardia* infection are patients who are immunocompromised. Nevertheless, patients with normal immunity accounted for one-third of all nocardiosis cases (2). Patients who have undergone organ transplantation, suffer from cancer, chronic nephrosis, or diabetes mellitus (DM) and those receiving long-term corticosteroids or immunosuppressants have an increased risk of acquiring *Nocardia* infection. The main clinical diseases afflicted with nocardiosis are pulmonary infection, sepsis, chronic bronchitis, brain abscess, skin abscess, and so on (3, 4). Half of the pulmonary nocardiosis cases are disseminated and also involve extrapulmonary infections, including pericardial, mediastinal, skin, subcutaneous tissue, and central nervous system (CNS) infections. Approximately 20% of the disseminated nocardiosis cases are entirely extrapulmonary diseases. Patients with normal immunity may primarily develop subcutaneous nocardiosis. The histopathological features of nocardiosis are abscess and obvious necrosis with neutrophil infiltration, which is usually surrounded by the granulation tissue, but extensive fibrosis or encapsulation is rare. More than 30 species of *Nocardia* have been reported to cause nocardiosis, and the most familiar species are *Nocardia asteroides, Nocardia nova, Nocardia farcinica*, etc. (5). Sulfonamides are still the first choice for the treatment of *Nocardia*. The combination of SMZ–TMP (sulfamethoxazole and trimethoprim) is equally effective or even more effective than SMZ alone, but the hematological toxicity of SMZ–TMP seems to be slightly higher.

## Materials and methods

This two-center retrospective study evaluated the data from adult patients who presented with *Nocardia* and had received treatment at the Zhongshan Hospital, Fudan University, Shanghai (Shanghai General Hospital) or at the Zhongshan Hospital, Fudan University, Xiamen branch (Xiamen branch) between January 2017 and May 2022.

We collected information about the adult cases of patients with *Nocardia* by extracting the field “*Nocardia*” in the Hospital Information Systems (HISs) of two hospitals. The diagnosis was confirmed by a microbiologically positive result, and those patients who were suspected but proved to be microbiologically negative were excluded. Finally, we obtained 19 targeted cases. Fifteen of these cases were from the Shanghai General Hospital and four of them were from the Xiamen branch. We described and analyzed the clinical characteristics of the nocardiosis cases from the following aspects: age and sex, *Nocardia* species, site of *Nocardia* infection, test specimens, detection methods, concurrent pathogens, symptoms, imaging features, co-conditions, drug susceptibility tests, antibiotic therapy/duration, outcomes, and follow-up. We described and analyzed the clinical characteristics of the 19 cases in terms of the median and quartile because of their non-normal distribution.

The studies involving human participants were reviewed and approved by the Ethics Committee of the Zhongshan Hospital, Fudan University, Shanghai, and Zhongshan Hospital, Fudan University, Xiamen branch. Written informed consent was obtained from the participants in our study.

## Results

### A two-center retrospective analysis

The demographic and baseline characteristics of the 19 cases are presented in Tables 1, 2. The median age of the 19 cases was 64 years, with an interquartile range (IQR) of 56–68 years. The age of the patients ranged from 15 to 80 years. Eleven out of 19 (57.9%) cases were men.

**Table 1:** Summary description of 19 cases from two hospitals.

**Case**	**Age (year)/** **sex**	***Nocardia* species**	**Sites of *Nocardia* infection**	**Test specimens**	**Detection methods**	**Concurrent pathogens**	**Symptoms**	**Imaging features**	**Coconditions**	**Drug susceptibility test (Y/N)**	**Antibiotic therapy/** **duration (month)**	**Outcomes**	**Duration of follow-up (month)**
1	80/M	*Nocardia*	Pulmonary	BALF	Bacterial culture	NTM, *Klebsiella pneumoniae*	Fever, cough, sputum	Consolidation	Pneumoconiosis, chronic bronchitis	Y	Clarithromycin, linezolid, carbapenem/6 months	Died 1 year after diagnosis	12
2	65/M	*N. brasiliensis*; *N. wallace*	Pulmonary	BALF	mNGS	None	Cough, sputum	Consolidation	SSA positive	N	SMZ, minocycline/13 months	Survived	56
3	49/F	*N. nova*	Pulmonary	Drainage of lung abscess	mNGS, bacterial culture	Aspergillosis	Cough, sputum	Cavity, mass, bronchiectasis	ABPA, RA; oral-corticosteroid, hydroxychloroquine, Iguratimod	Y	Amoxicillin, doxycycline/6 months	Survived	22
4	62/F	*Nocardia*	Pulmonary	BALF	Microbiological examination	None	Cough, fever, sputum	Bronchiectasis, infiltration, consolidation nodules	History of TB, bronchiectasis	N	Linezolid, SMZ/4 months	Survived	60
5	40/F	*Nocardia*	Pulmonary	Sputum	Bacterial culture	None	Fever, cough, sputum	Consolidation	Behcet's disease; oral-corticosteroid, MMF+AZA	Y	Carbapenem, minocycline/9 months	Survived	59
6	55/F	*Nocardia*	Pulmonary	Lung tissue, BALF	Bacterial culture	None	Cough, sputum, haemoptysis	Consolidation, bronchiectasis	Bronchiectasia	Y	Linezolid, minocycline/58 months	Survived	58
7	77/M	*Nocardia*	Pulmonary	Hydrothorax	mNGS	None	Chest pain, shortness of breath	Pleural effusion	None	N	Minocycline/14 months	Survived	52
8	61/M	*N. otitidiscaviarum*	CNS, Pulmonary	Blood, CSF	mNGS	Intestinal microsporidia	Fever, cough, sputum	Mass, cavity, pleural effusion	Membranous nephropathy; oral-corticosteroid, CTX	N	Carbapenem, SMZ/30 months	Survived	45
9	56/M	*N. farcinica*	Cutaneous, pulmonary, CNS	Pus, CSF	Bacterial culture	NTM	Fever, cough, sputum	Cavity, consolidation, nodules	Nephrotic syndrome	Y	Linezolid, moxifloxacin, imipenem/18 months	Died 18 months after diagnosis	18
10	15/M	*Nocardia*	Lumbar vertebra	Spinal cord	mNGS	Mycobacterium tuberculosis	Back pain	MRI: abscess, narrow of intervertebral space	None	N	SMZ, linezolid, meropenem/13 months	Survived	40
11	63/F	*N. abscessus complex; N. brasiliensis*	Pulmonary	Sputum, BALF	Bacterial culture, mNGS	CMV, EBV	Cough, sputum	Mass, bronchiectasis	COPD, bronchiectasia; ICS	Y	Doxycycline, levofloxacin/6 months	Survived	37
12	65/F	*Nocardia*	Pulmonary, CNS	Drainage of lung abscess	Bacterial culture	None	Weight loss	Mass, infiltration, nodules	SLE, DM; oral-corticosteroid	Y	Linezolid, SMZ, doxycycline/20 months	Survived	31
13	58/M	*Nocardia*	Pulmonary	Sputum	mNGS	Aspergillus	Cough, fever, haemoptysis, chest pain	Consolidation	None	N	Meropenem, amikacin/4 days; SMZ, doxycycline/1 month	Survived	24
14	46/F	*Nocardia*	Pulmonary	BALF, sputum	Bacterial culture	Klebsiella pneumoniae, pseudomonas aeruginosa	Cough, haemoptysis, cough, sputum	Cavity, bronchiectasis	None	Y	Doxycycline, SMZ/18 months	Survived	25
15	64/M	*Nocardia*	Pulmonary	Sputum	Bacterial culture	None	Shortness of breath, cough, chest pain, sputum.	Interstitial, bronchiectasis	Bronchiectasia, COPD and IPF; ICS, Pifefinidone	N	Cefatriaxone, SMZ/7 months	Survived	8
16X	73/M	*N. danyuensis*; *N. arcarensis*	Pulmonary	Sputum, BALF	Bacterial culture, mNGS	None	Cough, sputum	Bronchiectasis, interstitial	History of TB	N	SMZ/7 months	Survived	12
17X	44/F	*N. farcinica*	Pulmonary	BALF	Bacterial culture, mNGS	None	Right back pain	Consolidation	None	N	SMZ/7 months	Survived	7
18X	67/M	*N. brasiliensis*	Hand and wrist joints	Pus	mNGS	Trichophyton	Left hand pain	MRI: bone destruction, joint swelling	None	N	SMZ, minocycline/8 months	Survived	8
19X	65/M	*N. mastitis*	Pulmonary	Sputum, BALF, Blood	Bacterial culture, mNGS	Cryptococcus, stenotrophomonas maltophilia	Chest pain, cough, sputum	mass, infiltration, nodules	Membranous nephropathy; DM; oral-corticosteroid, FK506	Y	Minocycline, levofloxacin/3 months	Died 3 months after diagnosis	3

The No. 1–15 cases were from Zhongshan Hospital, Fudan University, Shanghai. 16X, 17X, 18X, and 19X were obtained from Zhongshan Hospital, Fudan University, Xiamen branch.

mNGS, metagenomic next-generation sequencing; BALF, bronchoalveolar lavage fluid; CSF, cerebrospinal fluid; CMV, cytomegalovirus; EBV, Epstein–Barr virus; SMZ, sulfamethoxazole; MMF, mycophenolate mofetil; AZA, azathioprine; CTX, cyclophosphamide; FK506, tacrolimus; CNS, central nervous system; SLE, systemic lupus erythematosus; DM, diabetes mellitus; NTM, nontuberculous mycobacteria; SSA, Sjogren syndrome A antibody; ABPA, allergic bronchopulmonary aspergillosis; RA, rheumatoid arthritis; TB, tuberculosis; COPD, chronic obstructive pulmonary disease; ICS, inhaled corticosteroid; IPF, idiopathic pulmonary fibrosis.

**Table 2:** Clinical characteristics of 19 cases of *Nocardia* infection.

**Clinical characteristics**	***N* = 19**
Age, years, median (IQR)	64 (56–68)
Men, *n* (%)	11 (57.9)
Underlying condition, *n* (%)	
Corticosteroid use	5 (26.3)
Immunosuppressant agent use	4 (15.8)
Diabetes mellitus	2 (10.5)
Chronic kidney disease	3 (15.8)
Chronic lung disease	7 (36.8)
History of tuberculosis	2 (10.5)
ICS	2 (10.5)
Symptom	
Fever	5 (26.3)
Cough and expectoration	14 (73.7)
Local pain	7 (36.8)
Shortness of breath	3 (15.8)
Haemoptysis	4 (21.1)
Site of clinical *Nocardia* infection, *n* (%)	
Pulmonary	17 (89.5)
Central nervous system	3 (15.8)
Cutaneous	1 (5.3)
Bone/joint	2 (10.5)
Antibiotic duration (months), median (IQR)	18 (13–20)
Antibiotic therapy, *n* (%)	
Single agent	3 (15.8)
Two concurrent agents	12 (63.6)
Three concurrent agents	4 (21.1)
Sulfonamide	11 (57.9)
Carbapenem	6 (31.6)
Linezolid	6 (31.6)
Aminoglycoside	1 (5.3)
Quinolone	3 (15.8)
Tetracyclines	11 (57.9)
β-lactam	2 (10.5)
Macrolide	1 (5.3)
Outcome, *n* (%)	
Overall mortality	3 (15.8)
Medication and following up	4 (21.1)
Improvement	12 (63.3)
Duration of follow-up (months), median (IQR)	45 (31–58)

Cough and expectoration (73.7%) was the most common symptom of nocardiosis at the time of presentation, followed by local pain (36.7%), fever (26.3%), hemoptysis (21.1%), and dyspnea (15.8%). The respiratory tract was the most common site of the clinical disease, which affected 84.2% of these patients. In 14 of these patients, only the lung was infected. Extrapulmonary disease was also common, as 2 cases had evidence of bone/joint involvement, 3 cases had central nervous system (CNS) involvement, 1 case had skin and soft tissue involvement, 1 case had lumbar vertebra involvement, and 1 case had hand and wrist joint involvement.

The comorbidities of these cases included diabetes (10.5%), chronic kidney disease (CKD) (15.8%), chronic lung disease (36.8%), and rheumatic diseases (10.5%). Only six patients had no underlying disease. Chronic lung disease (7 cases) was the most common underlying disease, which included bronchiectasis (3 cases), chronic bronchitis with postoperative lung abscess (1 case), allergic bronchopulmonary aspergillosis (ABPA, 1 case), chronic obstructive pulmonary disease (COPD) with bronchiectasis (1 case), and idiopathic pulmonary fibrosis (IPF) with bronchiectasia and COPD (1 case). Two patients had a history of pulmonary tuberculosis. Oral corticosteroids were used by five patients (26.3%) and immunosuppressants were used by four patients (21.0%). As detailed in Table 2, one patient with Behcet's disease received mycophenolate mofetil (MMF) and azathioprine (AZA), while two patients with membranous nephropathy were treated with cyclophosphamide (CTX) or tacrolimus (FK506). The case of rheumatoid arthritis (RA) was treated with long-term oral hydroxychloroquine and iguratimod. All patients with COPD received inhaled corticosteroids (ICS), while ABPA was treated with oral corticosteroids.

### Microbiology

The microbiological data of these 19 cases are presented in Table 3. More than half (*N* = 10, 52.6%) of the patients had concurrent infections with *Nocardia* at the time of presentation. Six out of ten patients had a concurrent bacterial infection (*Klebsiella pneumoniae, Pseudomonas aeruginosa, Mycobacterium tuberculosis, Nontuberculous mycobacterium*, and *Stenotrophomonas maltophilia*); one of the ten patients had a viral infection [cytomegalovirus (CMV) and Epstein–Barr virus (EBV)]; three out of ten patients had a fungal infection (*Aspergillus species, Cryptococcus* and *Trichophyton*); and one of the ten patients had an *intestinal microsporidial* infection. The *intestinal microsporidial* infection also affected the central nervous system (CNS). One case of non-tuberculous mycobacteria (NTM) infection disseminated to the skin, the hesoft tissues, the lungs, and the brain. The tuberculosis (TB) infection site was diagnosed with lumbar tuberculosis, and the *Trichophyton* infection site was the joint of the left hand. The remaining coinfected bacteria, fungi, viruses, NTM, etc., were all lung infections.

**Table 3:** Microbiological factors of 19 cases of *Nocardia* infection.

**Microbiological factors**	***N* = 19**
Detection method of *Nocardia, n* (%)	
Bacterial culture	10 (52.6)
mNGS	9 (47.4)
*Nocardia* detected in specimen, *n* (%)	
Blood	2 (10.5)
BALF and/or sputum	14 (73.7)
CSF	2 (10.5)
Pulmonary biopsy tissue	1 (5.3)
Bone	1 (5.3)
Hydrothorax	1 (5.3)
Pus	4 (21.1)
*Nocardia* species, *n* (%)	
*Nocardia brasiliensis*	3 (15.8)
*Nocardia farcinica*	2 (10.5)
*Nocardia abscessus* complex	3 (15.8)
Other	6 (31.6)
Not identified	10 (52.6)
Concurrent other pathogens (*N* = 10), *n* (%)	
*Aspergillus*	2 (20.0)
*Cryptococcus*	1 (10.0)
*Trichophyton*	1 (10.0)
*Non-tuberculous mycobacterium*	2 (20.0)
*Mycobacterium tuberculosis*	1 (10.0)
*Klebsiella pneumoniae*	2 (20.0)
*Pseudomonas aeruginosa*	1 (10.0)
*Stenotrophomonas maltophilia*	1 (10.0)
*CMV+EBV*	1 (10.0)
*Intestinal microsporidia*	1 (10.0)
Antimicrobial susceptibility (*N* = 9), *n* (%)	
Sensitive to drugs	8 (88.9)
Sulfonamide resistance	1 (11.1)

Other: other Nocardia species, 1 case of each species, including Nocardia wallace, Nocardia nova, Nocardia otitidiscaviarum, Nocardia danyuensis, Nocardia arcarensis, and Nocardia mastitis.

The most common site for the isolation of *Nocardia* species is the respiratory tract (73.7%), followed by local pus, cerebrospinal fluid (CSF), blood, the pulmonary biopsy tissue, the bone, and the hydrothorax. Invasive manipulation was the most common way to obtain microbial samples. The traditional diagnostic method of nocardiosis is bacterial culture. Metagenomic next-generation sequencing (mNGS) is a new approach that can detect the species of *Nocardia*. In our study, cultures in more than half of the cases (10/19) were unable to detect the *Nocardia* species. A total of 11.1% (*N* = 1) of the isolates in our cases were resistant to TMP–SMX, while no linezolid or any other drug resistance was described, as shown in Table 3.

### Imaging features

All 19 patients underwent chest CT (computed tomography). Bronchiectasis (*N* = 7) and consolidation (*N* = 7) were the most common presentations of the chest CT findings, followed by masses (*N* = 5), cavities (*N* = 4), nodules (*N* = 3), infiltration (*N* = 3), interstitial effusion (*N* = 2), and pleural effusion (*N* = 2).

**Figure 1:**
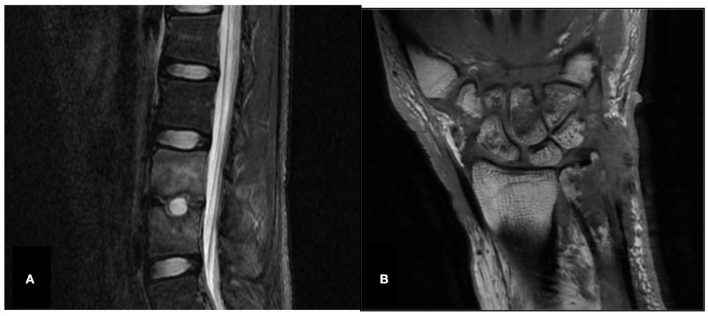
Spine and joint MRI (Case 10 and Case 18X). **(A)** Case 10, spinal magnetic resonance imaging (MRI), abnormal signals in the L3 and L4 vertebrae, abscess, narrowing intervertebral space and disappearance of the normal disc signal. **(B)** Case 18X, left wrist joint MRI, left distal ulna, multiple carpal and metacarpal bone destruction, left wrist joint soft tissue swelling.

The chest CTs of two patients were normal for only bones/joints. In one case, spinal magnetic resonance imaging (MRI) showed an abscess and narrowing of the intervertebral space. The joint MRI showed bone destruction and joint swelling in the other case (as shown in Figure 1).

**Figure 2:**
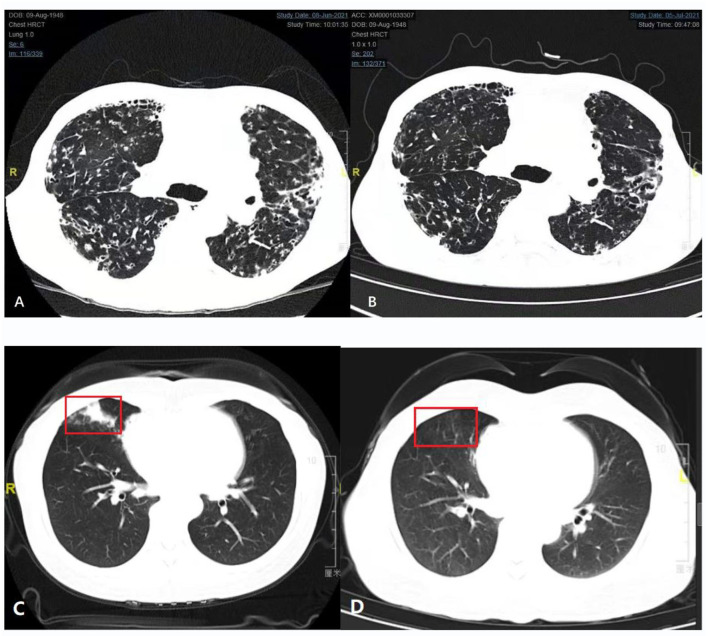
Comparison of chest computed tomography (CT) before and after treatment (Case 16X and Case 17X). **(A,B)** Comparison of chest computed tomography (CT) before and after 4 weeks of antibiotic treatment (Case 16X). **(A)** Plain chest CT scan before antibiotic therapy. **(B)** Plain chest CT scan after 1 month of antibiotic therapy. **(C,D)** Comparison of chest CT before and after 3 months of antibiotic treatment (Case 17X). **(C)** Plain chest CT scan before antibiotic therapy. The red box is a typical *Nocardia* infection site. **(D)** Plain chest CT scan after 3 months of antibiotic therapy. Inflammation of the red box had subsided.

We obtained CT images of cases 16X and 17X, whose imaging findings are shown in Figure 2. We compared the chest CT before and after 4 weeks of antibiotic treatment of case 16X in Figures 2A,B. In Figure 2A, the chest CT plain scan before antibiotic therapy showed local cystic and columnar dilatation of bronchi in both lungs. The surrounding lung tissue of the bronchi had multiple plaques and speckled images with high density. The adjacent pleura of the bronchi was thickened with multiple cystic lucid images in both lungs. In Figure 2B, the chest CT plain scan after 4 weeks of antibiotic therapy shows that some patchy and speckled lesions were absorbed, but the rest of the lung is similar to that before treatment.

We also compared the chest CT before and after 3 months of antibiotic treatment of case 17X in Figures 2C,D. Patient 17X was a 44-year-old woman who had no history of chronic lung disease or immunosuppression therapy. Before treatment, her lung CT showed consolidation of the middle lobe of the right lung, as shown in Figure 2C. After 3 months of antibiotic treatment, her CT scan of the chest revealed that the inflammation of the right lung had subsided, as shown in Figure 2D.

### Treatment and outcome

All patients were treated with antibiotic therapies, and 3 patients (15.8%) were treated under monotherapy. The majority of patients were subjected to treatment with two or more concurrent antimicrobial agents (63.6 and 21.1%, respectively). The main therapeutic drugs were tetracycline or SMZ (*N* = 11, 57.9%), followed by linezolid or carbapenems (*N* = 6, 37.6%). Thirteen patients discontinued the therapy; 12 out of 13 cases were due to stable disease, while the remaining 1 case was due to drug intolerance. Two patients (case 6 and case 14) were still receiving medication at the end of the follow-up period because of fever or coughing from time to time. Another two patients (cases 17X and 18X) were newly diagnosed and under therapy and were treated for 7 and 8 months, respectively. The median duration of treatment was 18 months (IQR 13–30 months). The all-cause mortality of patients with *Nocardia* infection was 15.8%. After a median follow-up of 45 months (IQR 31–58 months), 3 (15.8%) patients died (case 1, case 9, and case 19X).

The patient in case 1 was an 80-year-old man who had a history of structural lung disease. He had concomitant NTM infection and *Klebsiella pneumoniae*. The patient had a history of silicosis for 13 years and chronic bronchitis for more than 10 years. He received medication for 6 months due to a deteriorating physical condition and advanced age. Then, he died of multiple organ failure (MOF) and severe infection 1 year after *Nocardia* diagnosis.

The patient in case 9 had suffered from nephrotic syndrome for 20 years and was not given correct treatment. He had a wide spectrum of infections involving the thorax, the lungs, and the central nervous system (CNS). His concomitant NTM infection remained difficult to control. After 18 months of treatment for his *Nocardia* infection, he died of severe infection and MOF.

The patient in case 19X was a 65-year-old man whose underlying diseases were diabetes mellitus (DM) and membranous nephropathy with long-term oral corticosteroids and tacrolimus (FK506). Fever with respiratory failure led to his admission to the intensive care unit (ICU). Bronchoscopy and alveolar lavage were performed to confirm the diagnosis of nocardiosis and cryptococcosis. Despite active antifungal and anti-*Nocardia* treatment, he finally died of complex complications, renal insufficiency, hypokalaemia and septic shock 3 months after the diagnosis of nocardiosis.

## Discussion

This study is the largest nocardiosis case collection to date, with cases collected from two centers. *Nocardia* is a relatively rare opportunistic pathogen. Infection is caused by exogenous inhalation or by a direct invasion of the injured skin. The lung and skin are the most susceptible organs. Spine and joint infections are rare but not completely unknown. The characteristics of nocardiosis include abscess formation, extensive neutrophil infiltration, and significant necrosis (6). These pathological changes are similar to those of tuberculosis. People with immune deficiency are more susceptible.

The study of Weng et al. (7) showed that, when comparing the nocardiosis culture and next-generation sequencing (NGS) methods, the latter method can not only improve the detection rate of *Nocardia* but also greatly reduce the turnaround time. Nearly half of the patients in this study had *Nocardia* diagnosed by the mNGS method. The identification of the *Nocardia* species is important to guide treatment.

The drugs to treat *Nocardia* are sensitive to include TMP–SMZ, amikacin, linezolid, imipenem, and so on. Different *Nocardia* species have different drug susceptibility results. However, TMP–SMZ is the first choice for the treatment of *Nocardia* when the susceptibility test is positive. As its drug resistance rate to TMP–SMZ increased to 10.8%, imipenem and amikacin are good options (8). An *in vitro* study showed drug interactions between linezolid and amikacin, but a combination of these two agents should be avoided (9). Oral minocycline is well tolerated and can be used in patients with mild nocardiosis. For patients with severe diseases that cannot accept oral therapy or patients with SMZ-resistant strains, imipenem, ceftriaxone, cefotaxime, or amikacin are alternatives (10).

Oral therapy is the first choice for patients with no severe clinical diseases. Considering the resistance of *Nocardia* species to SMZ and minocycline, oral therapy, including linezolid, clarithromycin, amoxycillin clavulanate, or others, works well for the treatment of nocardiosis (10). Although linezolid is more commonly used to treat nocardiosis, several reports recommended amoxycillin clavulanate as an effective treatment when given in combination with other drugs (11–13). Clinicians should be aware of the higher rate of *Nocardia* resistance to TMP–SMZ and amikacin when treating these infections. Before treatment, drug sensitivity tests should be performed to develop a suitable therapeutic schedule.

According to published reports, the total duration of treatment (intravenous first, oral second) depends on the severity of the disease and the clinical and radiological response to therapy. The duration of intravenous antibiotic therapy should be 2–3 weeks for patients without CNS involvement and 3–6 weeks for those with CNS nocardiosis (14). Then, the patients should be switched to oral therapy when their clinical symptoms improve. Clinicians usually extend the duration of treatment to minimize the risk of disease recurrence. When infected with pulmonary or multifocal disseminated (non-CNS) *Nocardia*, the duration of treatment might extend by a duration of 6–12 months for patients with normal immunity. When the CNS is involved, the duration of treatment should be maintained for 12 months. At least 12 months of treatment are recommended for immunodeficient patients, regardless of the organ involved (15).

Magnetic resonance imaging (MRI) is an important method to determine the focus size and the infection situation in the brain, the spine, or the joint. MRI has a stronger soft tissue resolution than CT and is superior to CT in the visualization of brain, spinal cord, and intraarticular lesions. CT is an important method for the diagnosis of diseases, especially thoracic diseases. In the early stage of antibiotic treatment, if the clinical condition of patients with nocardiosis improves, a CT scan (4–6 weeks after initial drug administration) is helpful to evaluate the patient's response to antibiotic therapy. If the patient's clinical status does not improve, an early (2-week) CT scan is essential to evaluate the true state of the lesion and find the local cause of the negative results (16).

## Limitations

This study has very few limitations. One limitation of this study is that it pertains to the small sample size, as *Nocardia* is a rarely encountered opportunistic pathogen and cases of nocardiosis are infrequent. Also, this two-center study could not reflect the overall clinical characteristics of different *Nocardia* species in China.

## Conclusion

Nocardiosis is an uncommon life-threatening infection that affects patients suffering from structural lung disease or immunosuppression. Although nocardiosis is capable of progressing into a serious and metastatic disease, early recognition of the disease and prompt treatment measures given on time to the patient usually result in successful outcomes benefitting the patient.

## Data availability statement

The original contributions presented in the study are included in the article/supplementary material, further inquiries can be directed to the corresponding author/s.

## Ethics statement

The studies involving human participants were reviewed and approved by the Ethics Committee of Zhongshan Hospital, Fudan University, Shanghai and the Ethics Committee of Zhongshan Hospital, Fudan University, Xiamen. The patients provided their written informed consent to participate in this study. Written informed consent was obtained from the individual(s) for the publication of any potentially identifiable images or data included in this article.

## Author contributions

LW collected and processed data and wrote the introduction, materials and methods, and discussion. YX processed and analyzed data and wrote the results. ZC provided CT figures. WJ and XX provided consultation. YS collected data. YY provided idea. All authors contributed to the article and approved the submitted version.

## Funding

This work was supported by grants from Science and Technology Guidance Project of Xiamen (3502Z20214ZD1079), Xiamen Government and Xiamen Medical and Health Guidance Project (3502Z20199037), Xiamen Government.

## Conflict of interest

The authors declare that the research was conducted in the absence of any commercial or financial relationships that could be construed as a potential conflict of interest.

## Publisher's note

All claims expressed in this article are solely those of the authors and do not necessarily represent those of their affiliated organizations, or those of the publisher, the editors and the reviewers. Any product that may be evaluated in this article, or claim that may be made by its manufacturer, is not guaranteed or endorsed by the publisher.
