# Corrigendum: Nocardiosis: A two-center analysis of clinical characteristics

**DOI:** 10.3389/fmed.2023.1143851

**Published:** 2023-02-06

**Authors:** Lumin Wang, Yijiao Xu, Zhisheng Chen, Weiwen Jiang, Xiong Xiao, Yun Shen, Yanrong Ye

**Affiliations:** ^1^Xiamen Branch, Zhongshan Hospital, Fudan University, Xiamen, China; ^2^Zhongshan Hospital, Fudan University, Shanghai, China

**Keywords:** *Nocardia*, nocardiosis, clinical characteristics, immunosuppressed, two-center retrospective study

In the published article, there was an error in affiliation, 1. Instead of “Zhongshan Hospital, Fudan University, Xiamen, China,” it should be “Xiamen Branch, Zhongshan Hospital, Fudan University, Xiamen, China.”

The authors apologize for this error and state that this does not change the scientific conclusions of the article in any way. The original article has been updated.

